# Clustering Nuclear Receptors in Liver Regeneration Identifies Candidate Modulators of Hepatocyte Proliferation and Hepatocarcinoma

**DOI:** 10.1371/journal.pone.0104449

**Published:** 2014-08-12

**Authors:** Michele Vacca, Simona D'Amore, Giusi Graziano, Andria D'Orazio, Marica Cariello, Vittoria Massafra, Lorena Salvatore, Nicola Martelli, Stefania Murzilli, Giuseppe Lo Sasso, Renato Mariani-Costantini, Antonio Moschetta

**Affiliations:** 1 Fondazione Mario Negri Sud, Santa Maria Imbaro (Chieti), Chieti, Italy; 2 Unit of General Pathology, Aging Research Center (Ce.S.I.), “Gabriele D'Annunzio” University and Foundation, Chieti, Italy; 3 Interdisciplinary Department of Medicine, “Aldo Moro” University of Bari, Bari, Italy; 4 National Cancer Institute, IRCCS Oncologico “Giovanni Paolo II”, Bari, Italy; INRA, France

## Abstract

**Background & Aims:**

Liver regeneration (LR) is a valuable model for studying mechanisms modulating hepatocyte proliferation. Nuclear receptors (NRs) are key players in the control of cellular functions, being ideal modulators of hepatic proliferation and carcinogenesis.

**Methods & Results:**

We used a previously validated RT-qPCR platform to profile modifications in the expression of all 49 members of the NR superfamily in mouse liver during LR. Twenty-nine NR transcripts were significantly modified in their expression during LR, including fatty acid (peroxisome proliferator-activated receptors, *PPARs*) and oxysterol (liver X receptors, *Lxrs*) sensors, circadian masters *RevErbα* and *RevErbβ*, glucocorticoid receptor (*Gr*) and constitutive androxane receptor (*Car*). In order to detect the NRs that better characterize proliferative status *vs*. proliferating liver, we used the novel Random Forest (RF) analysis to selected a trio of down-regulated NRs (thyroid receptor alpha, *Trα*; farsenoid X receptor beta, *Fxrβ*; *Pparδ*) as best discriminators of the proliferating status. To validate our approach, we further studied PPARδ role in modulating hepatic proliferation. We first confirmed the suppression of PPARδ both in LR and human hepatocellular carcinoma at protein level, and then demonstrated that PPARδ agonist GW501516 reduces the proliferative potential of hepatoma cells.

**Conclusions:**

Our data suggest that NR transcriptome is modulated in proliferating liver and is a source of biomarkers and *bona fide* pharmacological targets for the management of liver disease affecting hepatocyte proliferation.

## Introduction

The liver is a major player in the modulation of lipid and glucose metabolism, xenobiotic detoxification, and is also responsible for serum protein synthesis. Under normal condition, mature hepatocytes represent up to 80% of hepatic cells, and are able to repopulate the liver upon different conditions, with a really slow turnover [1]. Even if cell division is rarely seen in hepatocytes of the normal adult liver [2], [3], differentiated hepatocytes show a remarkable replicative capacity after liver injuries [1], [4]. Liver regeneration (LR) is a compensatory growth of all mature functioning cells in the liver after different stimuli (e.g. hepatectomy, hepatocyte necrosis/apoptosis) [2], [5], [6]. Partial hepatectomy (PH) is considered a valuable model for studying in standardized conditions the complex mechanisms allowing hepatocyte proliferation, and for translating this knowledge in models of liver disease (e.g. chronic hepatitis and hepatocellular carcinoma, HCC). In rodents, PH consists in the removal of 60–70% of the liver mass (median and left lateral lobes) [7]. LR after PH is controlled by three clusters of networks: cytokines, growth factors and metabolic signals [2], [8]. After PH, 95% of the normally quiescent hepatocytes rapidly enter in the S phase of the cell cycle becoming able to replicate. This so called “*priming phase*” is mostly driven by inflammatory pathways (interleukin-6, IL-6; tumor necrosis factor alpha, TNFα; nuclear Factor-κB, NF-κB; signal transducer and activator of transcription 3, STAT-3; activator protein 1, AP-1; mitogen-activated protein kinase, MAPK), while the “*proliferative phase*”, during which hepatocytes proliferate restoring their original number, is under the control of several growth factors and, at intracellular level, of the Rb family member p107 and cyclins A, D, and E [2], [7]–[9]. The “*termination phase*”, during which regenerative process stops, occur within one week in rodents and is controlled by telomere length, transforming growth factor β (TGFβ), and interleukin-1β (IL-1β) [2], [7], [8].

Nuclear Receptors (NRs) are transcription factors transducing different signals into the modulation of gene activity [10]. NRs (48 in humans, 49 in rodents) are key players in the modulation of liver physiology and development, being also involved in cell growth and differentiation [10]. Some NRs are regulated by small lipophilic ligands (e.g. hormones, vitamins, dietary lipids, bile acids, and xenobiotics), while other NRs, namely “true orphans”, regulate transcription independently from binding to specific ligands [11]. NRs are suitable targets for pharmacological approaches aimed to the control of hepatocyte proliferation [12], since they may modulate a number of early changes essential for the liver regeneration and HCC, such as the activation of transcription factors [AP-1; NF-κB; STAT3; and CCAAT/enhancer binding protein (C/EBP) beta], and the expression of immediate early genes [FBJ murine osteosarcoma viral oncogene homolog (c-Fos); jun proto-oncogene (c-Jun); v-myc avian myelocytomatosis viral oncogene homolog, c-Myc; liver regenerating factor 1, LRF-1; early growth response 1, EGR-1] cytokines and growth factors [13]–[21]. In addition, many NR ligands can induce hepatocyte proliferation also in the absence of liver injury (i.e. “direct hyperplasia”) [21], [22]. This is the case of fibrates (agonists of the peroxisome proliferators activated receptors alpha, Pparα), thyroid hormones, and halogenated hydrocarbon TCPOBOP (agonist of the constitutive androstane receptor, Car) [22], [23].

The aim of our study was to analyze the changes of NR transcriptome in liver regeneration after PH to generate a cluster of NRs changes characterizing proliferating liver, in order to understand the involvement of NRs in the pathophysiology of liver regeneration, and to find candidate biomarkers and putative targets for the management of liver disease. To support the relevance of the NRs cluster analysis in identifying novel targetable hits to modulate hepatocyte proliferation, we activated PPARδ pharmacologically using its high-affinity synthetic agonist GW501516, and we showed that PPARδ reduces the proliferative rates of Hepa 1-6 hepatoma cell line.

## Materials and Methods

### Animals

C57BL/6 wild type mice were hosted under a standard 12 hr light/12 hr dark cycle and fed with standard rodent chow and water *ad libitum*. 10–12 week old male mice were used for the experiments. All the animal protocols were approved by the Ethical Committee of the Fondazione Mario Negri Sud. PH was performed according to the method of Higgins and Anderson under ketamine/xylazine anesthesia [15], [24]. The left lateral and median lobes were completely excised. For the sham-operated controls, an excision was made into the peritoneal cavity, and the liver was exteriorized and put back into the peritoneal cavity followed by closure of the incision. Mice (4–5 per group) were sacrificed at different time points after hepatectomy (day 0, 0.5, 1, 3, & 7). Liver integrity was assessed with the serum levels of alanine transaminase (ALT) and aspartate transaminase (AST), as markers of liver injury. Data were normalized to day 0 at each time point after PH. To measure the fraction of hepatectomy, the livers were excised from each groups of mice, their weights were compared to the initial total liver mass calculated from the total body weight of each animal [15], [20].

### RNA extraction and reverse-transcription

Total RNA was isolated by QIAzol Lysis Reagent (Qiagen) following manufacturer's instructions. To avoid possible DNA contamination, RNA was treated with DNAase-1 (Ambion, Foster City, CA). RNA purity was checked by spectrophotometer, while RNA integrity was assessed by Biorad Experion. Only samples with Relative Quality Index (RQI)>8 were used for reverse-transcription. According to the manufacturer's instructions, cDNA was synthesized by reverse-transcribing 4 µg of total RNA using the High Capacity DNA Archive Kit (Applied Biosystem).

### Quantitative real-time Polymerase Chain Reaction (RTqPCR)

RTqPCR primers were designed using Primer Express software and previously validated and published [25]. PCR assays were performed in 96 well optical reaction plates using the ABI 7500HT system (Applied Biosystem). PCR assays were conducted in triplicate wells for each sample. Baseline values of amplification plots were set automatically and threshold values kept constant to obtain normalized cycle times and linear regression data. The following reaction mixture per well was used: 5 µl Power Sybr Green (Applied Biosystem), 1.2 µl primer at the final concentration of 150 nM, 0.8 µl RNAse free water, 3 µl cDNA (30 ng). For all experiments the following PCR conditions were used: denaturation at 95°C for 10 min, followed by 40 cycles at 95°C for 15 seconds, then at 60°C for 60 seconds. Individual receptor PCR efficiencies were calculated from the slope of the resulting standard curves, using the formula E = 10^−1/slope^ where E is efficiency. Indeed, the obtained efficiency was used to convert cycle times from log to linear scale using the formula E^−ct^. Normalized mRNA levels were expressed as relative units and were obtained by dividing the averaged, efficiency-corrected, values for NR mRNA expression by that of glyceraldehyde-3-phosphate dehydrogenase (*Gapdh*) as internal controls. The resulting values were multiplied by 10^6^ for graphical representation and plotted as mean ± SEM [25]. Analyses of NRs expression profiling were performed based on the example of the anatomical profiling of NRs expression by *Bookout et al* [25], [26]. The relative units used to define the mRNA expression levels were obtained from the formulas above assuming a Ct>35 for absent, 35<Ct<30 for low, 30<Ct<25 for moderate and Ct<25 for high expression [25], [26]. In LR experiments, normalized mRNA expression levels were defined as: absent if the relative units were below 0.1, low if between 0.1 and 8.9, moderate if between 8.9 and 1324 and high if above 1324.

### Cell Culture

Hepa1-6 cells obtained from the American Type Culture Collection (ATCC) were maintained at 37°C in 5% CO2 in Dulbecco's Modification of Eagle's Medium (DMEM) with 10% fetal bovine serum (FBS), and 1% penicillin/streptomycin (P/S). Hepa 1–6 cells were plated in 6-well plates at density of 2×10^5^ cells/well for cell cycle and microarray experiments. After overnight seeding, we performed a 24 h serum starvation, then cells were maintained in fresh DMEM medium containing 10% FBS and 1% P/S, and treated with dimethyl sulfoxide (control) or GW501516 (Santa Cruz Biotechnology, Cat. sc-202642A) at a concentration of 10 µM. This concentration of GW501516 was previously used and validated by other groups, being shown to specifically activate PPARδ [19], [27], [28]. After 48 h treatment, we quantified cells, extracted RNA/proteins, and studied cell cycle.

### Western Blot

Cells were homogenized in RIPA (Sigma-Aldrich) lysis buffer with protease inhibitors cocktail (Roche) and phosphatase inhibitor cocktail (Sigma-Aldrich). The lysates were kept on ice for 30 min and then centrifuged at 10000 g at 4°C for 10 min. Protein concentration was determined by the Bradford method (Bio-Rad Laboratories) in order to load the same amount (30 µg) of total proteins. Proteins were separated on a 10% sodium dodecyl sulfate–polyacrylamide gel and transferred onto a nitrocellulose membrane. Membranes then were blocked with 5% BSA in 0.05%, Tris-buffered saline–Tween-20, and probed with specific antibodies [anti-proliferating cell nuclear antigen (Pcna), Santa Cruz Biotechnology, Santa Cruz, CA; anti-phospho-Stat3, Cell Signaling, Danvers, MA; anti- heat shock protein 90 -HSP90-, BD Bioscience]. Membranes finally were incubated with horseradish-peroxidase - conjugated secondary antibodies (anti-rabbit, Calbiochem, Darmstadt, Germany). The signal was detected using the ECL-enhanced chemiluminescence system (Amersham, Piscataway, NJ).

### Fluorescence-Activated Cell Sorter Analysis of the Cell Cycle

Hepa 1-6 cells were fixed in 70% ethanol and stained for 1 hour with propidium iodine. Cell-cycle distribution was measured with a FACS Vantage flow cytometer (BD Bioscience, Milan, Italy) and analyzed by using Cell Quest-PRO software (BD Bioscience). At least 20000 events per sample were acquired. Cell-cycle analysis was performed using ModFit LT 3.0 software (Verity Software House, Topsham, ME).

### Microarray analysis for gene expression profiling in Hepa 1-6 after GW501516 treatment

Microarray gene expression analysis was conducted on RNA extracted from the HEPA 1-6 48 h after GW501516 treatment. Whole RNA (400 ng) was used for cRNA synthesis using the Illumina Total Prep RNA Amplification kit (Ambion, Austin, TX, US) following the manufacturer's instructions. Whole-Genome gene expression experiments were conducted using the Illumina whole genome direct hybridization assay (MouseRef-8 v2.0 Expression Bead-Chips) on the Illumina microarray platform (Illumina iScan System). Upon the manufacturer instructions, data were processed using the Illumina Genome Studio Software through specific algorithms of filtration and cleaning of the signal. Data were normalized together with the quantile method. Background was not subtracted. Final output consisted of normalized fluorescence intensity of each probe (AVG signal), representing the expression levels of each gene. AVG signal lower/equal to the background and with detection p value>0.001 was excluded. We excluded genes discontinued or poorly annotated in NCBI Entrez Gene Database records. We thus performed pathways analysis on a final number of 48 significant genes (Fold>1.3; p<0.05 according to the “Illumina custom” error model) using the “Core Analysis” function of Ingenuity Pathway Analysis (Ingenuity System Inc., USA) to identify networks associated with GW501516 stimulation.

### Histology and Immunohistochemistry

Mice tissue specimens were fixed in 10% formalin for 12–24 hours, dehydrated and paraffin embedded. We also performed immunohistochemistry on samples of paraffin-embedded HCC (*vs.* paired normal tissues; n = 9) received from Creative Bioarray, USA (http://www.creative-bioarray.com/Contact-Us.html). Standard Immunohistochemistry protocols were performed [15]. Briefly, 5 µm-thick sections were treated with 3% hydrogen peroxide for 5 min and with the Dako Cytomation Biotin blocking system (Dako, Denmark) to quench endogenous peroxidase and biotin respectively. Sections were sequentially incubated for 60 min at room temperature in 50% non-immune serum in PBS (to avoid unspecific signals) and overnight at 4°C with the primary antibody (anti-Pcna, Santa Cruz Biotechnology, Santa Cruz, CA; Abcam Anti-PPAR delta antibody, Cat AB23673). Sections were then washed for 10 min in PBS, and incubated for 30 min at room temperature with the secondary biotinylated antibody (Vector Laboratories). After several washing steps with PBS (3 washes 5 min/each), sections were incubated with the avidin-biotin complex (Vector Laboratories) for 30 min at room temperature. After washing in PBS, the peroxidase reaction was initiated by incubation with DAB (Sigma-Aldrich, Milano, Italy). Coverslips were mounted with Permount and evaluated under a light microscope. All the stained sections were analyzed through a confocal microscope (Magnitude: 20×). For each sample, 5 representative images were taken. Number and intensity of marked nuclei were quantified using *ImageJ* software as previously described [29].

### Statistical analysis

All the data were first analyzed with classical statistical approaches to evaluate differences among groups, and correlations between clinical and prognostic variables and levels of expression of specific NRs. In particular, the difference among multiple groups was assessed using the Mann-Whitney, Wilcoxon Signed-Rank Test, or the Kruskal Wallis test followed by post-hoc analysis (Nemenyi-Damico-Wolfe-Dunn test), when appropriated. These initial methods allowed ranking NRs according to p-values. Data were presented as means ± SEM. To find a correlation between continuous variables the Pearson's correlation coefficient was used. P-values<0.05 were considered statistically significant.

In order to detect the NRs that better discriminate among groups (proliferative status *vs*. proliferating liver), the more recent and innovative RF Analysis [30] was applied as a complement of the canonical approaches. The advantages of RF in dealing with gene selection and classification are well documented [31], [32]. A RF is a classification algorithm consisting of an ensemble of tree-structured classifiers and represents a highly accurate technique that overcomes the problem of low number of observations [31], [32]. The important features of RF are the identification and classification of relevant differentially expressed genes [31], and the estimation of the error rate related to their predictive ability. This efficient approach gave us the possibility to obtain a ranking of genes according to the variable importance measure (namely Relative Importance, RI, listed in **Table S1 in File S1**) and to define an “identity card” of genes characterizing the proliferating status.

According to RF analysis, 100,000 trees were built to classify tissues. The learning set used to grow each tree was a 632+ bootstrap resample of the observations; this means that about one-third of the cases were left out of the sample. Trees were allowed to grow to their full size without pruning. Each node was split using the best among a random subset of genes. The left-out observations (i.e. “out of bag” observations) were then predicted to obtain the classification error rate of the considered tree. Predictive ability of the algorithm was assessed aggregating the single tree error rates. This corresponds to an internal validation. Therefore, the advantage is that RF makes unnecessary a second external test set to get an unbiased estimate of the error. The estimation of RI was obtained by looking at how much the classification error increases (the C-index decreases) when “out of bag” data for that variable are permutated while all others are left unchanged. The importance metric used was the Mean Decrease in Accuracy (MDA). The MDA is constructed by permuting the values of each variable of the internal test set, recording the prediction and comparing it with the un-permutated test set prediction of the variable. After obtaining a ranking of genes based on descending order of RI, the best classifiers were identified according to some “elbow strategy” on the graph of their measure of importance. We followed *Strobl et al.* [33] to avoid possible bias in variable selection; individual classification trees were built using subsampling without replacement, and adopting a conditional permutation scheme [34].

All the analyses were performed using the SAS Package (Release 9.1) and the R Package (Version 2.12.2).

## Results

### Nuclear receptors in normal liver

**Figure 1:**
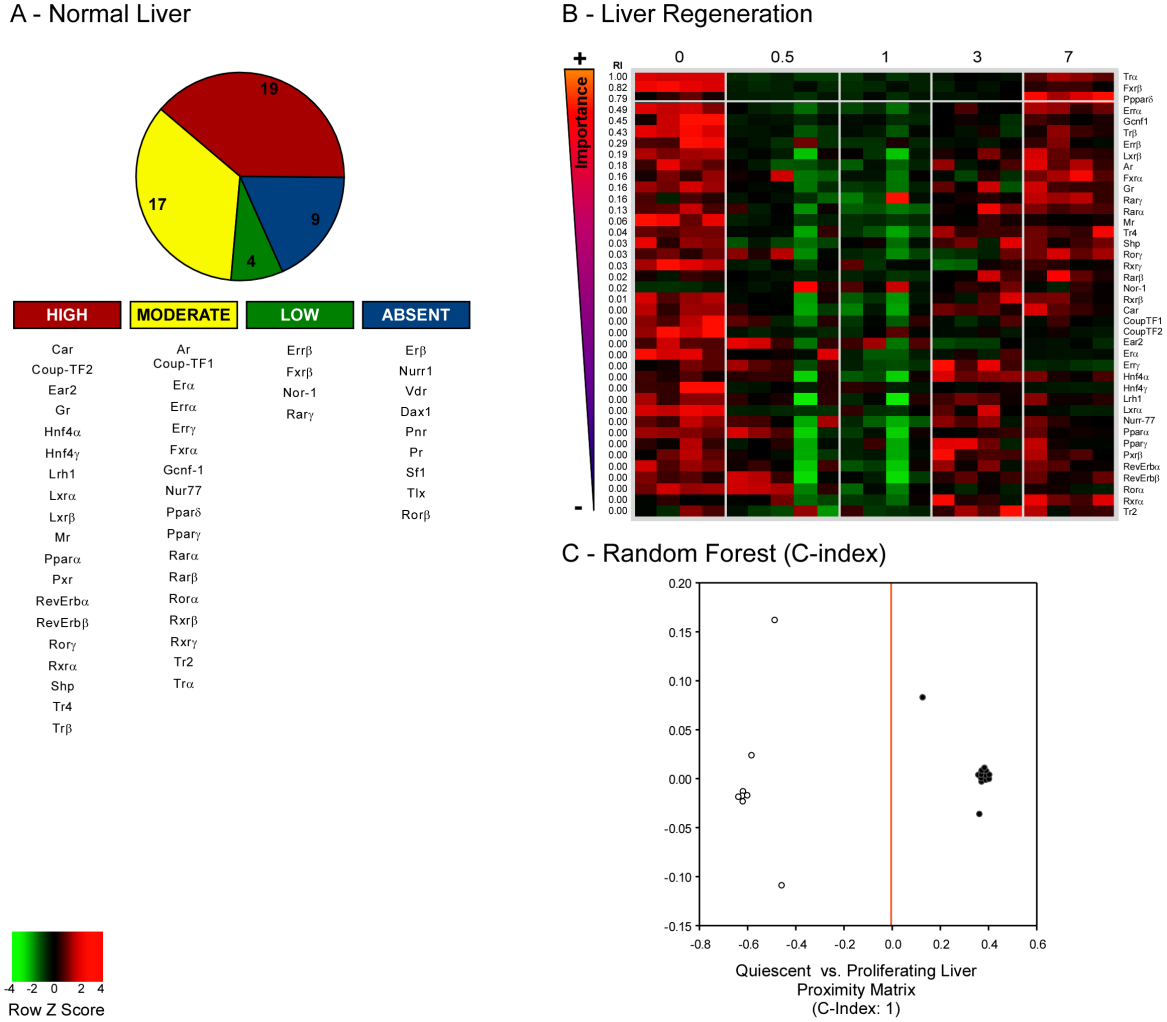
NRs mRNA expression levels in normal mouse liver (A) and after PH (B). Forty NRs were expressed in quiescent liver: 19 genes expressed at high level (red; Ct<25 or RU>1324), 17 NRs at moderate level (yellow; 25<Ct<30 or 8.9<RU<1324), 4 NRs at low level of expression (green; 30<Ct<35 or 0.1<RU<8.9), while 9 NRs were unexpressed (blue; Ct>35 or RU<0.1). Heatmap of the changes in NR transcriptome listed in order of RI at RF analysis, to identify candidate biomarkers of proliferation after PH. RF analysis highlights *Trα*, *Fxrβ* and *Pparδ* as classifiers of the “proliferative” status in LR experiments. Details of the changes observed for each NR are shown in Figure 2. (**C**) Proximity matrix of the RF algorithm. On the basis of the mRNA expression levels of *Trα*, *Fxrβ* and *Ppparδ*, RF discriminates a “quiescent” status (control liver and 7 days after PH) from a “proliferative” one (12 hours, 1 and 3 days after PH) in 100% of cases (C-Index = 1). *Gapdh* was used as reference gene, and values are expressed as relative units.

We first show the expression levels of each NR in normal liver, thus clustering NRs on the basis of their mRNA abundance (**Figure 1A**). Nineteen NRs were expressed at high mRNA concentration (i.e. Ct<25; RU>1324), 17 genes were expressed at middle concentration (i.e. 25<Ct<30; 8.9<RU<1324), 4 genes were expressed at low concentration (i.e. 30<Ct<35; 0.1<RU<8.9), while 9 genes were almost absent (i.e. Ct>35; RU<0.1; or completely unexpressed). The high mRNA expression level of numerous NRs in normal liver highlights the central role of NR driven pathways in liver physiology.

### Changes in nuclear receptor transcriptome during LR

**Figure 2:**
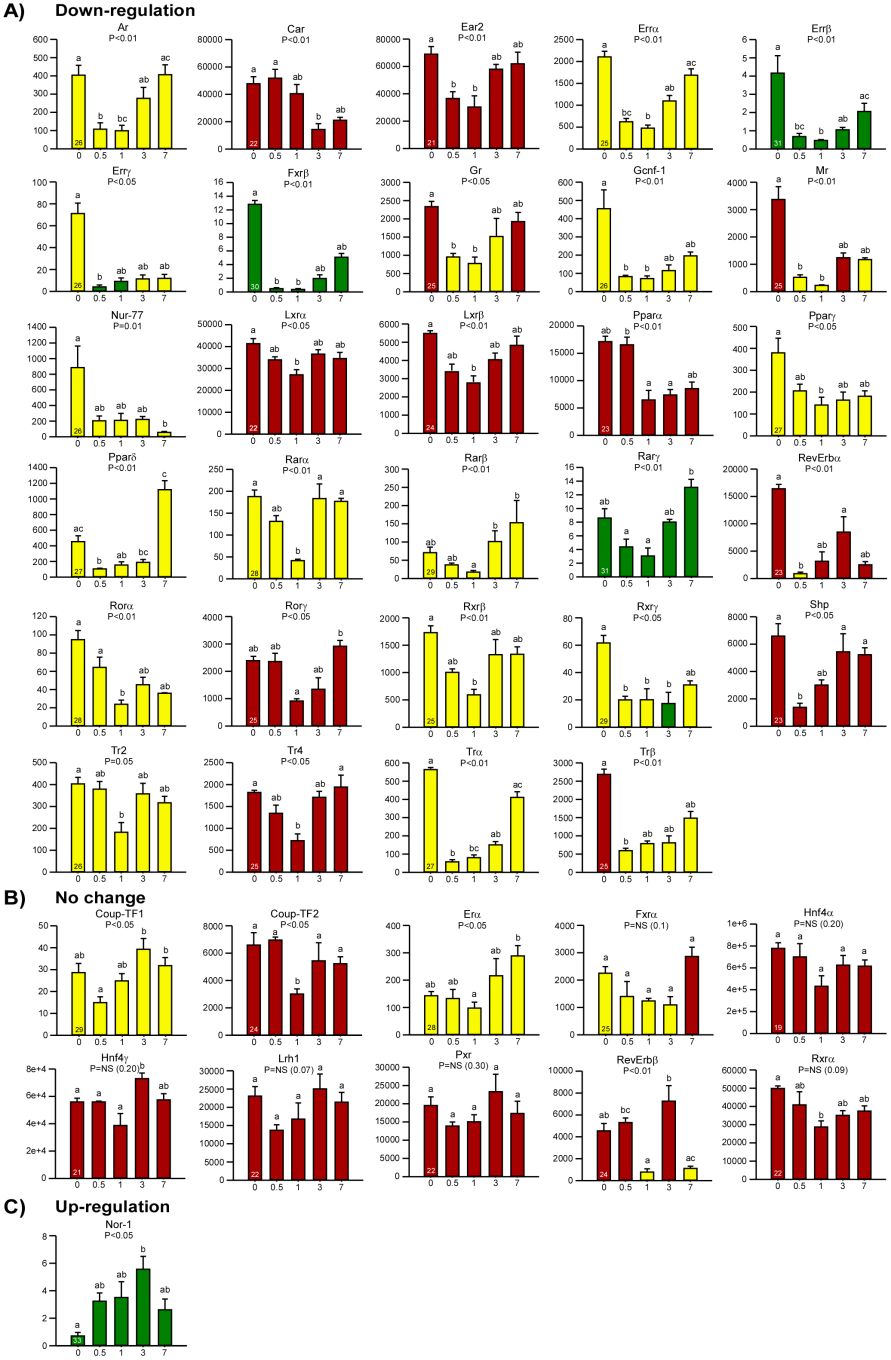
Detailed patterns of NR mRNA expression during LR in mice. (**A**) After PH, 29 of the 40 NRs expressed in the liver were down-regulated during the priming/proliferative phases of LR, with mRNA expression levels comparable to those of the control liver seven days after PH; (**B**) 10 NRs did not change significantly during LR; (**C**) 1 gene showed an increased mRNA expression pattern during the priming and proliferative phases, with mRNA expression levels comparable to those of the control liver seven days after PH. Unexpressed genes (*Erβ, Dax-1, Nurr-1, Pnr, Pr, Sf-1, Tlx, Rorβ, Vdr*) are not shown. The colors of the columns reflect the expression patterns shown in Figure 1A. *Gapdh* was used as reference gene, and values were expressed as relative units. Cycle time numbers at RTqPCR are reported in the “Day 0” bar. All the results are shown as mean ± SEM. Lower case letters indicate statistical significance (p≤0.05), assessed by the Kruskal-Wallis One-Way ANOVA on Ranks plus Nemenyi-Damico-Wolfe-Dunn post-hoc test (n = 4–5 at each time point); “a” means reference group; “b” means different from “a”; “a, b” means equal to both “a” and “b”; “c” means different from “a” and “b”; “a, c” means equal to both “a” and “c” and different from “b”; “b, c” means equal to both “b” and “c” and different from “a”.

In order to depict the changes in the NRs transcriptome of the proliferating liver, we performed a two-third partial hepatectomy in wild-type mice. Seven days after PH, we observed a complete regrowth of the liver (**Figure S1A**). Increased plasma ALT and AST levels were documented during the first 24 h of LR (**Figure S1B**); this event was followed by an increase of *c-Myc* and Cyclin E1 (*Ccne1*) transcripts, Pcna transcripts and staining, and a reduction of the *Tgfβ1* transcript during the priming and proliferation phases of LR (**Figure S1 C-F**). All these indicators of hepatocyte proliferation returned to normal values once liver mass was restored. We also found that 29 of the total 49 NRs were significantly down-regulated during the priming/proliferative phases of LR, while 10 were unchanged (**Figures 1B** & **2**). The only NR significantly increased during the proliferative stages was the orphan NR neuron derived orphan receptor 1 (*Nor-1*) [35]. The NRs signature 7 days after PH was statistically comparable to that of quiescent liver. Several NRs (i.e. androgen receptor, *Ar*; ERBA-related gene-2, *Ear2*; estrogen-related receptors, *Err α/β/γ; Fxrβ*; germ cell nuclear factor 1, *Gcnf-1*; mineralocorticoid receptor, *Mr; Pparδ; RevErbα*; retinoid X receptor gamma, *Rxrγ*; small heterodimer partner, *Shp; Trα*/*Trβ*) were characterized by significant and early (i.e. 12 hours after PH) modifications, while others (i.e. *Car*; glucocorticoid receptor, *Gr*; liver X receptors, *Lxr α/β; Nor-1; Ppar α/γ*; retinoid acid receptors, *Rar α/β/γ*; RAR-related orphan receptor alpha, *Rorα; Rxrβ*; testicular receptors, *Tr2 and Tr4*) displayed significant changes mainly in the proliferating stages (1 and/or 3 days) after PH (**Figure 2** & **Table S1 in File S1**). These data confirm a major modulation of the NR transcriptome in proliferating hepatocytes, when compared to quiescent liver.

### Changes in mRNA expression levels of *Trα*, *Fxrβ*, and *Pparδ* characterize liver regeneration

**Figure 3:**
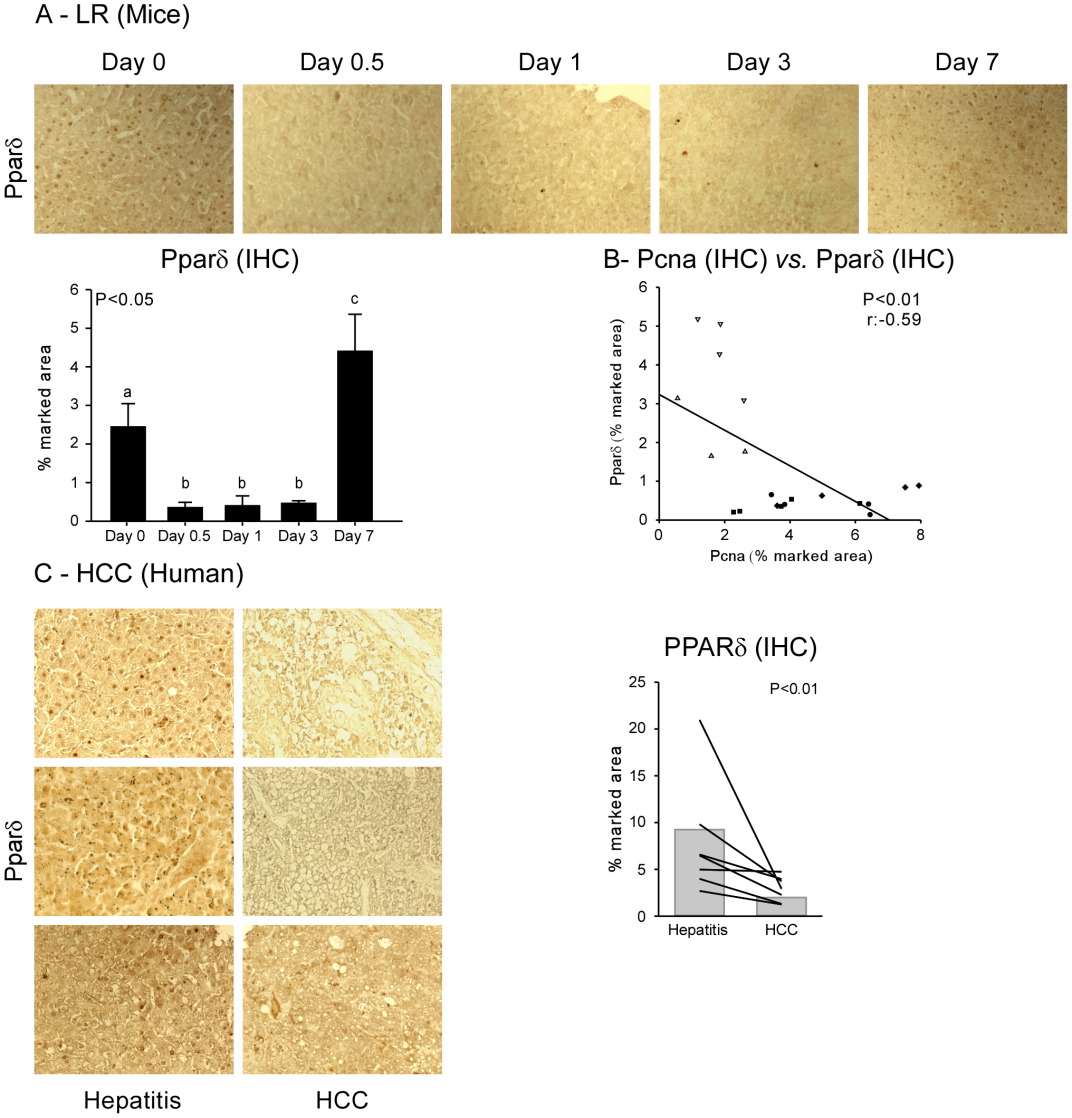
PPARβ/δ protein is suppressed in murine regenerating liver (A) and human HCC (C), and negatively correlates with PCNA (B). Anti-PPARβ/δ immunostaining in samples of LR (4–5/group) and 9 paraffin-embedded samples of HCC paired with reference tumor-free tissues (selection: 3 of 9 subjects); PPARβ/δ positive cells (percentage of marked area) were quantified using the ImageJ software. Results are shown as means ± SEM or means (bars) and cases (each line representing paired tumor-free tissue *vs*. tumor sample). Statistical significance (p≤0.05) assessed by the Kruskal-Wallis One-Way ANOVA on Ranks plus Nemenyi-Damico-Wolfe-Dunn post-hoc test (LR), Wilcoxon Signed-Rank Test (HCC), and Pearson's correlation coefficient (correlation). Legend: lower case letters indicate statistical significance (“a” means reference group; “b” means different from “a”; “c” means different from “a” and “b”); (white) quiescent; (black) proliferating; (▴) Sham; (▪) 0.5 days; (•) 1 day; (♦) 3 days; (▾) 7 days

Since there were no differences in the NRs transcriptome between control livers and those 7 day after PH (when liver regrowth was complete), we clustered these two time points in the definition of the “quiescent status”, while considering “proliferating status” the other time-points (i.e. 12 hours, 1, and 3 days after PH). We then analyzed our data with the novel RF analysis to highlight the best discriminators (in order of RI) of the two conditions (**Figure 1B**). We found as best discriminators of the proliferative status *Trα*, *Fxrβ*, and *Pparδ*. Hence, we checked the ability of these 3 genes to act as candidate biomarkers of proliferation, using another feature of the RF analysis. In fact, the RF algorithm allows the study of the discrimination ability of a specific set of genes as discriminators, and tests the power of this prediction for new samples (internal controls). On the basis of the levels of expression of *Trα*, *Fxrβ* and *Pparδ*, RF was able to discriminate “quiescent” from “proliferating” liver in 100% of cases (C-index = 1, see proximity matrix, **Figure 1C**). These data confirm that the changes in *Trα*, *Fxrβ* and *Pparδ* mRNA expression levels could represent the identity card of proliferating cells, thus highlighting these NRs as candidate biomarkers of liver proliferation, and as potential targets for novel pharmacological approaches. Finally, we studied Pparδ at protein level, showing that Pparδ protein expression was reduced in the liver during the regenerative phases that follow PH, and negatively correlated to Pcna staining (**Figure 3A–B**).

### Markers of cell proliferation (*Ccne1*, *cMyc, and Pcna*) and “growth termination” (*Tgfβ1*) correlate with levels of mRNA expression of the top three hits at RF analysis (*Trα, Fxrβ, Pparδ*)

**Figure 4:**
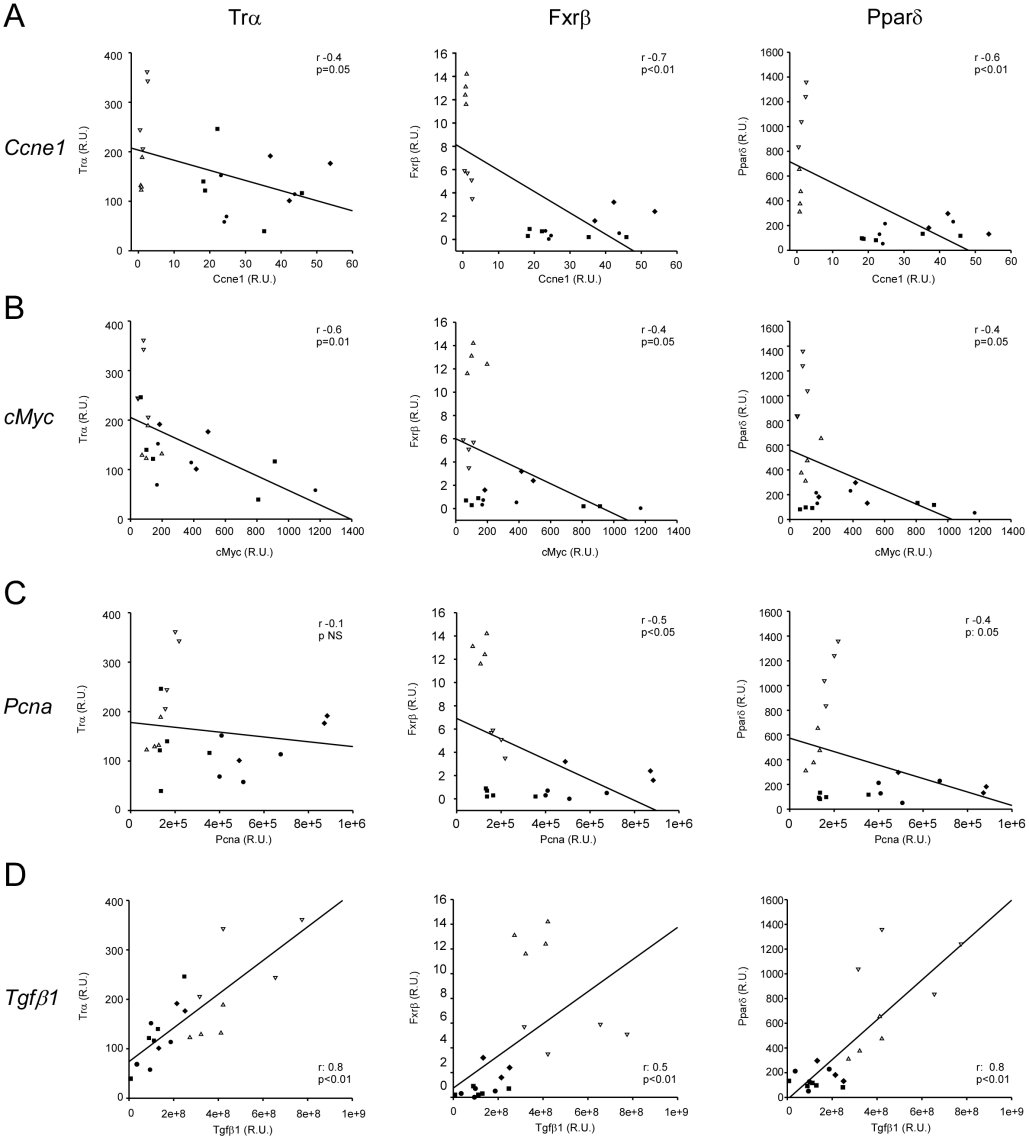
Markers of hepatocyte proliferation and growth termination correlate with the top three NRs at RF analysis. *Trα*, *Fxrβ*, and *Pparβ/δ* mRNA expression levels correlate negatively with *Ccne1* (**A**) and *cMyc* (**B**), and positively with *Tgfβ1* (**D**); *Fxrβ* and *Pparβ/δ*, but not *Trα*, also correlate negatively with *Pcna* expression levels (**C**) (0.4>Pearson's correlation coefficient<−0.4; p<0.05). Legend: (white) quiescent; (black) proliferating; (▴) Sham; (▪) 0.5 days; (•) 1 day; (♦) 3 days; (▾) 7 days.

Since *Trα, Fxrβ* and *Pparδ* were significantly down-regulated during the proliferative stages of LR and identified as best discriminators of the proliferating status at RF analysis, we thus tested if the mRNA expression levels of these NRs could be correlated to known markers of cell proliferation (levels of mRNA expression of *Ccne1*, *cMyc, and Pcna*) and of termination phase (*Tgfβ1*). Interestingly, *Trα, Fxrβ* and *Pparδ* correlated negatively with *Ccne1 and cMyc* and positively with *Tgfβ1*; *Fxrβ* and *Pparδ*, but not *Trα*, also correlated with Pcna transcript levels (**Figure 4**), underscoring the close relationship between these NRs with the PH-induced LR pathways, and further confirming these NRs as suitable targets for therapy aimed in modulating cell proliferation.

### PPARδ is suppressed in human HCC and its pharmacological activation reduces hepatoma cells growth

**Figure 5:**
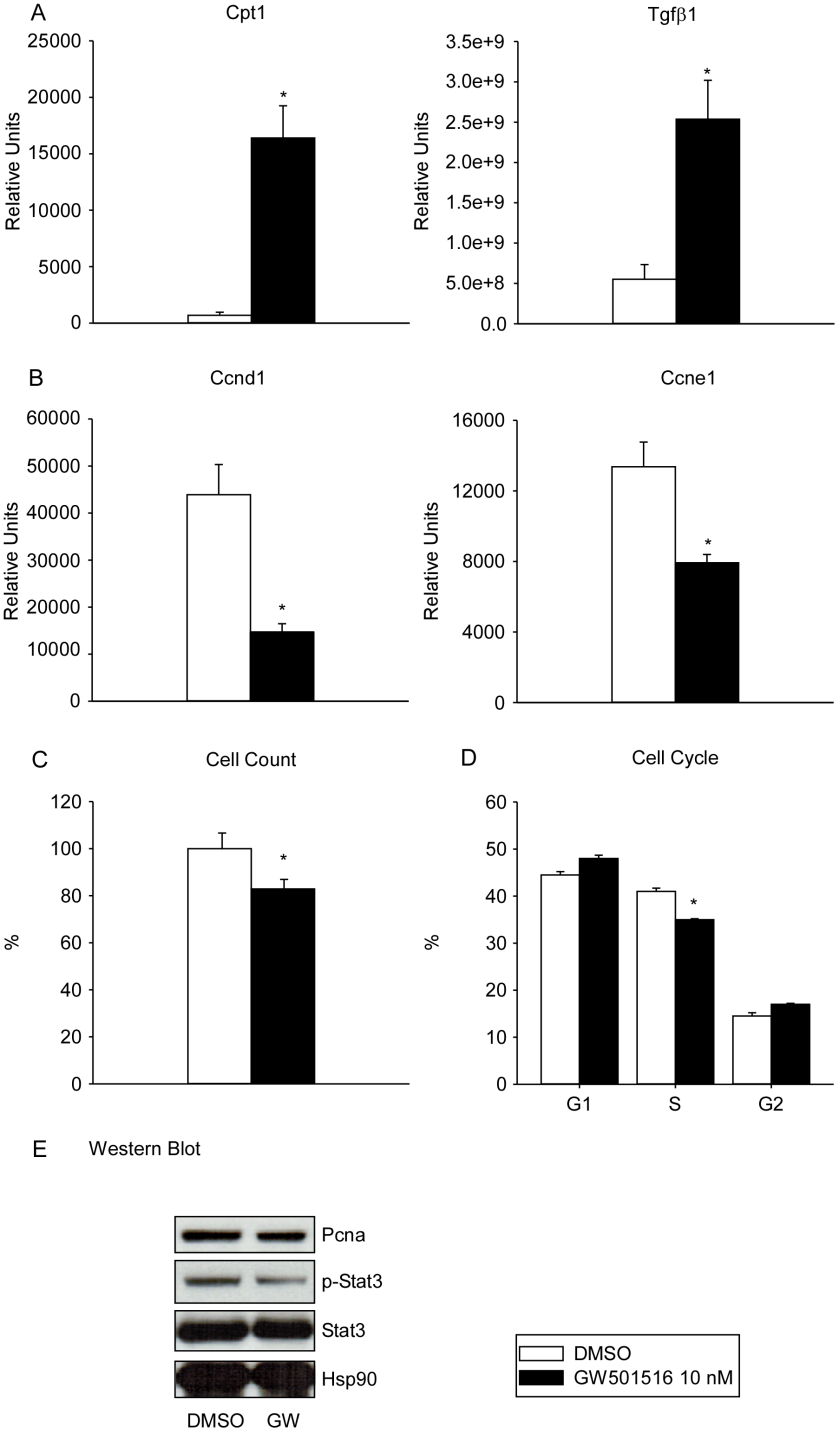
PPARβ/δ agonist GW501516 reduces Hepa 1-6 proliferation. Hepa 1–6 cells were treated with PPARβ/δ agonist GW501516 (10 nM) for 48 h. The efficacy of GW501516 in activating Pparβ/δ was confirmed at the mRNA level (**A**, relative mRNA expression of the Pparβ/δ target genes *Cpt-1* and *TGFβ1*). When compared to the DMSO treated cells, the proliferative response of Hepa 1–6 treated with GW501516 was reduced, as confirmed by relative mRNA expression of *Ccnd1* and *Ccne1* (**B**), cell count (**C**), S-Phase at the analysis of the cell cycle (**D**), Pcna protein expression and Stat-3 phosphorylation (**E**, Western blot). For mRNA expression, *Gapdh* was used as reference gene, and values were expressed as relative units. All the results are shown as mean ± SEM. Asterisks indicate statistical significance (p≤0.05), assessed by the unpaired Mann-Whitney rank sum test (triplicate/group).

In order to give a translational relevance to our findings generated in the murine model of hepatocyte proliferation, we studied if Pparδ protein was reduced in human HCC (*vs.* paired tumor-free tissue), and we found that Pparδ protein was significantly reduced in the human model of neoplastic growth (**Figure 3C**). The contribution of PPARδ in hepatocyte proliferation and HCC is still unclear and strongly discussed in literature; PPARδ knock out animals are characterized by a delay in LR exclusively in the early phases of LR (no data for late time points) [36], while PPARδ activation in HepG2 cells has been shown to both promote [19], [27], [37] and inhibit [28] cell proliferation, or even to have no influence [38]. To test the hypothesis that PPARδ could reduce the proliferative capacity of cancer cell lines, we administered GW501516 (10 nM) to proliferating hepatoma cells (i.e. Hepa 1–6). GW501516 induced the mRNA expression of PPARδ targets carnitine palmitoyltransferase 1 (*Cpt1*) and *Tgfβ1* [39] (**Figure 5A**), while suppressed cyclins D1 (*Ccnd1*) and *Ccne1* (**Figure 5B**). GW501516 negatively modulated Hepa 1–6 cell proliferation, as shown by a reduction of cell count and S phase cell growth curve (**Figure 5C–D**), a decreased of Pcna and Stat3 phosphorylation (**Figure 5E**, Western blot). Microarray analysis (**Table 1** **& Table S2 in File S1**) showed that PPARδ activation in Hepa 1–6 is associated to the up-regulation of genes involved in the modulation of the cell cycle and a suppression of genes involved in cancer development and progression. In detail, we identified the up-regulation of the pro-apoptotic caspase 8 (CASP8) [40], the tumor suppressors P67/methionyl aminopeptidase 2 (P67/MetAP2) [41], pyruvate dehydrogenase [lipoamide] kinase isozyme 4 (PDK4) [42] and protein angiopoietin-like 4 (ANGPTL4) [43] (which is suppressed in HCC when compared to perilesional tissue [44]), and of different genes known to be suppressed in HCC (i.e. CD82 [45]; WNT antagonist prickle homolog 1, PRICKLE1 [46]) and other neoplasms (i.e. DAZ associated protein 2, DAZAP2 [47]). On the other hand, PPARδ activation in Hepa 1–6 induces the down-regulation of polymerase delta 1 catalytic subunit (Pold1, negatively correlated to the prognosis of HCC patients [48]), zinc finger and BTB domain containing 7A (Zbtb7a, promoter of cancer cells proliferation [49], [50]), and Dual specificity phosphatase 7 (Dusp7, known to be over-expressed in cancer [51]).

**Table 1:** Networks modulated by PPARδ activation in proliferating Hepa 1–6 cells *in vitro*.

**Networks UP**	**Molecules in Network**
Modulation of Cell Cycle, Lipid Metabolism, Small Molecule Biochemistry	ACAA2, ACADL, Acot1, ANGPTL4, CASP8, CCT5, CD82, CHMP5, DAZAP2, IDI1, LPCAT3, LPL, METAP2, NUDCD2, PDK4, PRICKLE1, TFAM, TSG101, VBP1
Organismal Development, Lipid Metabolism, Small Molecule Biochemistry	CYP51A1, DGAT2, ECM1, ELOVL6, ETFDH, FCGRT, HSD17B12, IFRD1, MEMO1, PGRMC1, PRUNE
**Networks Down**	**Molecules in Network**
Cancer, Gastrointestinal Disease, Organismal Injury and Abnormalities	BACH1, DUSP7, NFATC3, PCYT1A, POLD1, TCOF1, ZBTB7A

These data suggest that PPARδ activation could be able to negatively modulate cell proliferation of Hepa 1–6 cells.

## Discussion

The knowledge of the complex networks promoting hepatocyte proliferation in different conditions is needed for a better understanding of the pathophysiology of chronic liver disease and hepatic carcinogenesis. In addition, no effective therapy is available for promoting hepatocyte renewal after hepatic injury, delaying the progression of chronic hepatitis to cirrhosis, and preventing the development and/or progression of HCC.

Mature hepatocytes are characterized by a remarkable replicative capacity [4]–[6], [52]. PH is controlled by three clusters of networks (cytokines, growth factors, and metabolic signals) cooperating to induce hepatocyte “priming”, “proliferation” and growth “termination” phases [5], [8]. Hence, the knowledge of mechanisms controlling these three main phases of LR is of great importance for characterizing the pathophysiology and the management of liver disease.

LR after PH thus represents a valuable model for studying mechanisms allowing hepatocytes proliferation, as well as the metabolic adaptive changes occurring in the liver after an injury [2], [9]. The adaptive response of the liver during regeneration tries to fulfill the metabolic needs of the body via the promotion of gluconeogenic response and glucose secretion [53], [54]. On the other hand, plasma triglyceride and cholesterol levels significantly decrease due to a dramatic increase of lipid/cholesterol uptake and utilization in the liver [55]. Hepatic cholesterol neo-synthesis is also induced when exogenous cholesterol has become insufficient to meet the cellular demand (e.g. cell membranes) [15], [56]. Many other adaptive responses occur as the result of different sets of transcription factors being differentially modulated [57], [58], thus determining peculiar LR-specific hepatic functions during LR (e.g. changes in the secretion of liver-specific proteins and enzymes, temporary suppression of hepatic functions, etc.) [2],[9]. In this view, studying NR transcriptome changes during LR could be a step forward in understanding the complex metabolic events underlying hepatocyte proliferation after PH.

The NR superfamily is a set of transcription factors acting as conductors of differentiated liver functions. NRs are master transcriptional regulators of different homeostatic processes (e.g. development, cell differentiation, metabolism, proliferation, and apoptosis), and can be modulated by different signals (e.g. hormones, vitamins, lipids) [59]. NRs are also implicated in LR modulation [15], [20], [21], and in the pathophysiology of liver disease [59]. We developed an atlas of NRs transcriptome in liver regeneration after PH to uncover the involvement of the NRs transcriptome in the modulation of LR, and to highlight a novel set of players in LR potentially acting as candidate biomarkers of LR and targets for modulating hepatic proliferation. We found a significant reduction of the overall NRs transcriptome during the priming and the proliferative phases of LR, while the NRs expression patterns were similar in the “growth termination” phase to those observed in the quiescent liver. In particular, we found a significant down-regulation of fatty acid and oxysterol sensors (i.e. *Pparα*, *Pparγ*, and *Lxrs*). Interestingly, a down-regulation of these NRs has been observed in a model of hepatic inflammation induced by lipopolysaccharide administration [60], supporting a possible involvement of the inflammatory pathways in the modulation of NRs expression and activity. A marked decrease of Pparα, Pparγ, and Lxr mRNA/function has been already documented in rodents after PH [12], [61]. In line with this, the activation of these NRs via synthetic ligands results in delayed LR, due to a reduced hepatic lipid and oxysterol contents, and not to a direct modulation of canonical LR signaling pathways [15], [17], [62]–[65]. We could also confirm an early (Day 0.5–1) and transient down-regulation of *RevErbα* and *RevErbβ* [66]. These changes can be connected to a previously described modulation of the circadian clock activity in proliferating hepatocytes [67]. *Nor-1* is the only up-regulated NR during LR. In this respect, we have recently shown that *Nor-1* is over-expressed also in human hepatocellular carcinoma and that Nor-1 knock-down blunts the regenerative capacity of the liver, while Nor-1 over-expression in normal liver induces a proliferative switch in differentiated liver with a mechanism independent from the canonical inflammatory pathways [35]. Nor-1 subfamily member nuclear receptor-related factor 1 *(Nurr-1)*, which is almost absent in the liver (cycling times >35, thus not shown in this atlas), is increased as well after PH [68], highlighting a role of the NR4A subfamily in the modulation of liver regeneration and hepatocyte proliferation.

We did not document changes in *Fxrα*, estrogen receptor alpha (*Erα*), pregnane X receptor (*Pxr*) and *Rxrα* transcripts. On the other hand, previous studies described changes in the activity of these NRs after PH; these NRs have been associated to the promotion of hepatocyte proliferation and LR, while the deletion of these genes is associated to defective LR [20], [69]–[72]. Additionally, we found *Gr* decreased at mRNA level. The role of Gr in the modulation of hepatocyte proliferation and hepatocarcinogenesis is discussed in literature. In humans and rats, GR seems to promote hepatocyte proliferation [73], [74] but, on the other hand, lack of GR is associated to enhanced hepatocyte proliferation and HCC development in mice [75]. At present, due to the large use of glucocorticoids in clinical practice, a better understanding of the molecular pathways underlying GR activation in hepatocyte proliferation is crucial, and needs to be further addressed.

Additionally, we found a down-regulation of *Car* in the later proliferative stages of LR (Day 3), and of *Trα* and *Trβ* in late priming stage (Day 0.5). These NRs are known to induce hepatocyte proliferation and direct hyperplasia, through mechanisms mediated primarily by Cyclin D1 [20]–[22], [76]. Probably, the PH-driven network acts independently to Car/Tr driven proliferative pathways (alternative to the growth factor/cytokine pathways) to induce hepatocyte proliferation [77], [78]. All the other observed changes in the present survey are novel, and their meaning need to be addressed in more comprehensive studies.

RF analysis allowed us to highlight, in order of importance, the NRs most consistently modified in proliferating liver (i.e. *Trα*, *Fxrβ* and *Pparδ*). RF is considered a highly accurate classifier, characterized by many decision trees and outputs. The algorithm allows detecting the discrimination ability of a specific dataset and tests if the decision tree is able to define a satisfactory prediction for a new sample. The changes in *Trα*, *Fxrβ* and *Pparδ* mRNA abundance classify the proliferating (i.e. 12 hours, 1 day and 3 days after PH) from quiescent liver (i.e. control livers and livers 7 days after PH) in 100% of cases (C-Index of 1 at RF analysis). Additionally, this trio of NRs is negatively and statistically correlated with known markers of hepatocyte proliferation (Pcna staining, *Ccne1* and *c-Myc*). These data underscore the putative active transcriptional role of NR in the complex mechanisms underlying hepatocyte proliferation.

We thus pointed to PPARδ down-regulation to validate our approach, with the aim of understanding if PPARδ suppression is confirmed also in HCC, and if it could negatively modulate hepatoma cells growth. PPARδ is a promising target since is a major player in the control of metabolic pathways modulating LR (glucose and fatty acid metabolism) [79], exerts an anti-inflammatory activity [80], have been involved in the modulation of cell proliferation and carcinogenesis [19], and can be modulated pharmacologically. The contribution of PPARδ in hepatocyte proliferation and HCC is strongly discussed in the literature; PPARδ knock out animals are characterized by delayed LR exclusively in the early phases of LR, but no data are available for time points later than three days (when PPARδ KO liver weight/body weight ratio are comparable to wild type mice) [36]. While PPARδ pharmacological activation in HepG2 cells has been shown to promote [19], [27], inhibit [28], or to be not influent [38] on cell proliferation depending on the experimental condition (for example in absence of serum, PPARδ activation inhibits proliferation [38]). Here we show that PPARδ protein is reduced in the murine experiments of LR and in human HCC, and that the activation of PPARδ in Hepa1-6 cells is able to inhibit proliferation.

We conclude that NRs are key actors in the modulation of liver function, and could be also actively involved in the regulation of LR and carcinogenesis. In this work, we showed that the NR transcriptome is profoundly modified in proliferating hepatocytes. Combining classical gene expression approaches with innovative algorithm classifier analyses, we were able to depict an “identity card” of LR after PH and to identify *Trα*, *Fxrβ* and *Pparδ* as candidate biomarkers and putative targets for the pharmacological modulation of LR, with a potential application in liver disease and HCC.

## Supporting Information

Figure S1 **Liver regeneration after PH.** (**A**) Percentage of initial liver weight at different time points after hepatectomy (PH); Serum concentration of the transaminase ALT and AST (**B**) at different time points after PH; (**C**) Pcna expression [Relative mRNA expression levels *Pcna* and Anti-Pcna immunostaining (percentage of Pcna positive cells calculated using ImageJ)] of proliferating liver at different time-points after partial hepatectomy. Relative mRNA expression levels of *Ccne1* (**D**), *cMyc* (**E**), and *Tgfβ1* (**F**) in regenerating liver, measured by RT-qPCR. For RT-qPCR, *Gapdh* was used as reference gene and values were expressed as relative units. All the results are shown as mean ± SEM. Lower case letters indicate statistical significance (p≤0.05), assessed by the Kruskal-Wallis One-Way ANOVA on Ranks plus Nemenyi-Damico-Wolfe-Dunn post-hoc test (n = 4-5 at each time point); “a” means reference group, “b” means different from “a”; “a, b” means equal to both “a” and “b”; “c” means different from “a” and “b”; “a, c” means equal to both “a” and “c”; “d” means different from “a”, “b”, and “c”; “e” means different from “a”, “b”, “c”, and “d”.
(TIF)

File S1 **Supporting tables.**
(DOC)
