# Supplementary figures and images for: Clustering Nuclear Receptors in Liver Regeneration Identifies Candidate Modulators of Hepatocyte Proliferation and Hepatocarcinoma
Source: PLoS One. 2014 Aug 12;9(8):e104449. doi: 10.1371/journal.pone.0104449 (PMC4130532; doi:10.1371/journal.pone.0104449)

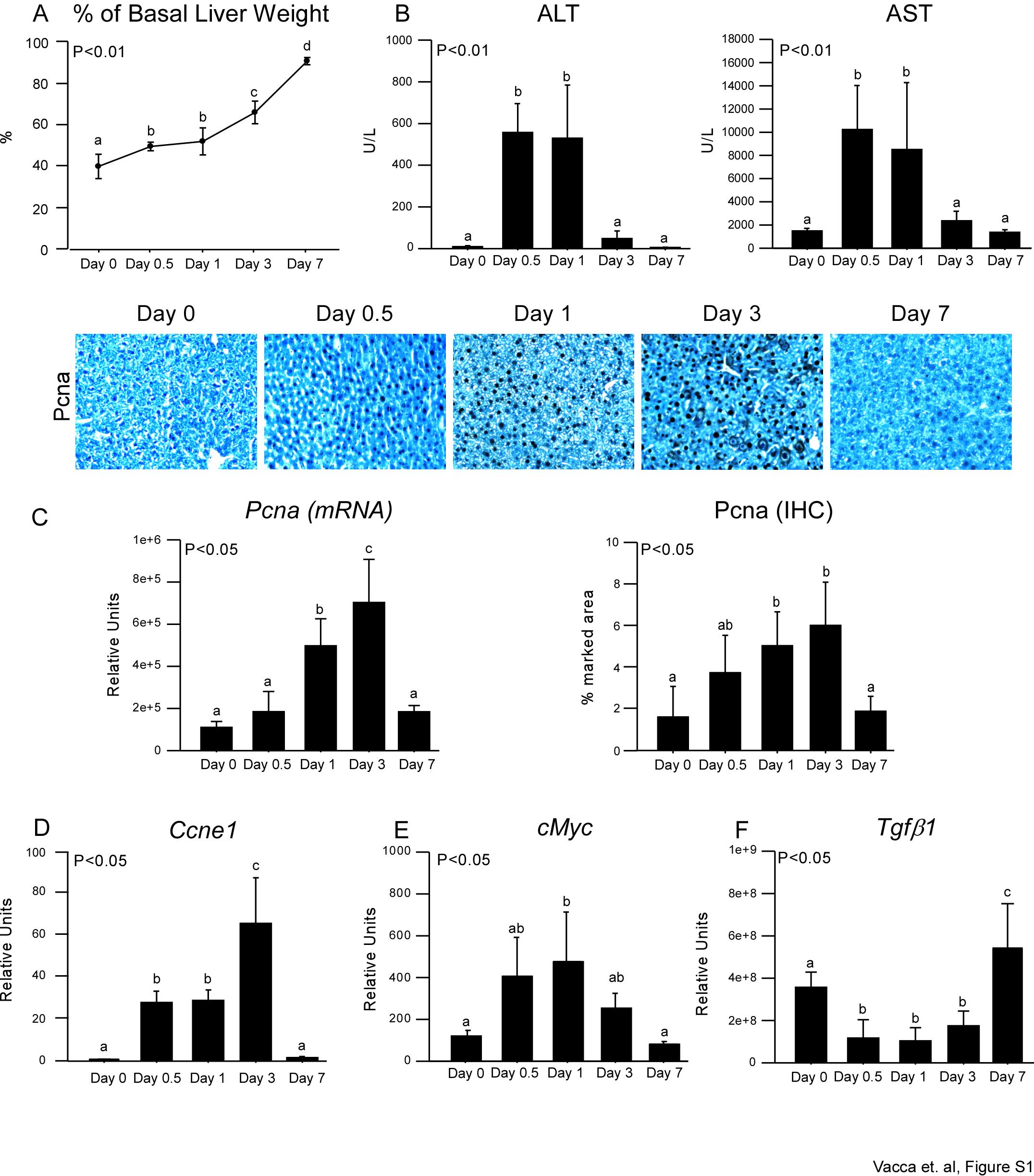

Supplement: Figure S1 — Liver regeneration after PH. (A) Percentage of initial liver weight at different time points after hepatectomy (PH); Serum concentration of the transaminase ALT and AST (B) at different time points after PH; (C) Pcna expression [Relative mRNA expression levels Pcna and Anti-Pcna immunostaining (percentage of Pcna positive cells calculated using ImageJ)] of proliferating liver at different time-points after partial hepatectomy. Relative mRNA expression levels of Ccne1 (D), cMyc (E), and Tgfβ1 (F) in regenerating liver, measured by RT-qPCR. For RT-qPCR, Gapdh was used as reference gene and values were expressed as relative units. All the results are shown as mean ± SEM. Lower case letters indicate statistical significance (p≤0.05), assessed by the Kruskal-Wallis One-Way ANOVA on Ranks plus Nemenyi-Damico-Wolfe-Dunn post-hoc test (n = 4-5 at each time point); “a” means reference group, “b” means different from “a”; “a, b” means equal to both “a” and “b”; “c” means different from “a” and “b”; “a, c” means equal to both “a” and “c”; “d” means different from “a”, “b”, and “c”; “e” means different from “a”, “b”, “c”, and “d”. (TIF) [file pone.0104449.s001.tif]
