# Supplementary material for: Clustering Nuclear Receptors in Liver Regeneration Identifies Candidate Modulators of Hepatocyte Proliferation and Hepatocarcinoma
Source: PLoS One. 2014 Aug 12;9(8):e104449. doi: 10.1371/journal.pone.0104449 (PMC4130532; doi:10.1371/journal.pone.0104449)
Supplement: File S1 — Supporting tables. (DOC) [file pone.0104449.s002.doc]

**Table S1. Detailed list of the levels of mRNA expression of each NR in the LR experiments.**

|  | **0** | | **12h** | | **1d** | | **3d** | | **7d** | | **ANOVA** | **Post Hoc** | | | | | | | | | |
| --- | --- | --- | --- | --- | --- | --- | --- | --- | --- | --- | --- | --- | --- | --- | --- | --- | --- | --- | --- | --- | --- |
|  | **Mean** | **SEM** | **Mean** | **SEM** | **Mean** | **SEM** | **Mean** | **SEM** | **Mean** | **SEM** | **P** | **0.5 vs. 0** | **1 vs.0** | **3 vs. 0** | **7 vs. 0** | **1 vs. 0.5** | **3 vs.0.5** | **7 vs. 0.5** | **3 vs.1** | **7 vs.1** | **7 vs.3** |
| **Mr** | 3369.53 | 469.09 | 523.74 | 91.96 | 228.73 | 21.14 | 1240.33 | 173.70 | 1172.33 | 58.48 | <0.01 | <0.05 | <0.01 | NS | NS | NS | NS | NS | 0.06 | 0.1 | NS |
| **Trα** | 563.15 | 6.87 | 57.38 | 12.05 | 80.33 | 15.01 | 150.50 | 18.14 | 410.90 | 27.81 | <0.01 | <0.01 | <0.05 | NS | NS | NS | NS | <0.05 | NS | 0.2 | NS |
| **Errα** | 2105.43 | 127.96 | 621.76 | 75.00 | 476.70 | 69.57 | 1096.88 | 126.17 | 1686.25 | 130.32 | <0.01 | <0.01 | <0.01 | NS | NS | NS | NS | 0.09 | NS | <0.05 | NS |
| **Fxrβ** | 12.83 | 0.55 | 0.46 | 0.14 | 0.38 | 0.15 | 1.98 | 0.54 | 5.05 | 0.49 | <0.01 | <0.01 | <0.01 | NS | NS | NS | NS | 0.08 | NS | 0.09 | NS |
| **Pparδ** | 453.43 | 74.81 | 104.42 | 9.30 | 154.48 | 41.21 | 188.00 | 37.87 | 1118.65 | 103.08 | <0.01 | <0.05 | NS | NS | NS | NS | NS | <0.01 | NS | <0.05 | NS |
| **Errβ** | 4.15 | 0.96 | 0.68 | 0.17 | 0.48 | 0.05 | 1.08 | 0.13 | 2.05 | 0.41 | <0.01 | <0.01 | 0.00 | NS | NS | NS | NS | 0.20 | NS | <0.05 | NS |
| **Rorα** | 94.73 | 10.04 | 64.38 | 11.21 | 23.85 | 4.59 | 45.28 | 8.21 | 35.90 | 0.53 | <0.01 | NS | <0.01 | 0.20 | NS | <0.05 | NS | NS | NS | NS | NS |
| **Trβ** | 2686.75 | 143.15 | 593.38 | 67.14 | 787.18 | 72.54 | 808.18 | 191.55 | 1484.15 | 166.22 | <0.01 | <0.01 | 0.06 | 0.08 | NS | NS | NS | 0.08 | NS | NS | NS |
| **Lxrβ** | 5482.25 | 148.36 | 3213.38 | 292.93 | 2693.63 | 270.35 | 4040.50 | 363.87 | 4826.03 | 455.26 | <0.01 | NS | <0.01 | NS | NS | NS | NS | NS | NS | NS | NS |
| **REVErbβ** | 4544.03 | 673.44 | 5308.16 | 414.29 | 778.98 | 293.94 | 7261.98 | 1417.09 | 1113.80 | 176.83 | <0.01 | NS | 0.10 | NS | NS | <0.05 | NS | 0.20 | <0.01 | NS | <0.05 |
| **Pparα** | 17124.10 | 993.28 | 16547.36 | 1405.44 | 6468.15 | 1747.25 | 7381.40 | 996.81 | 8532.63 | 1074.53 | <0.01 | NS | 0.06 | 0.07 | NS | <0.05 | <0.05 | 0.10 | NS | NS | NS |
| **Gcnf1** | 454.68 | 103.25 | 82.18 | 7.23 | 71.43 | 14.92 | 115.08 | 31.42 | 195.90 | 18.84 | <0.01 | <0.01 | <0.05 | 0.20 | NS | NS | NS | 0.10 | NS | 0.1 | NS |
| **Rarγ** | 8.63 | 1.36 | 4.38 | 1.13 | 3.08 | 1.15 | 8.00 | 0.40 | 13.08 | 1.04 | <0.01 | NS | 0.20 | NS | NS | NS | NS | <0.05 | NS | <0.05 | NS |
| **REVErbα** | 16402.63 | 801.79 | 874.90 | 297.00 | 3152.60 | 1718.05 | 8482.00 | 2814.62 | 2514.20 | 508.58 | <0.01 | <0.01 | 0.10 | NS | NS | NS | <0.05 | NS | NS | NS | NS |
| **Ear2** | 68986.73 | 5567.92 | 36555.72 | 4958.56 | 30315.45 | 8211.72 | 57998.00 | 3518.80 | 61925.03 | 7607.97 | <0.01 | <0.05 | 0.01 | NS | NS | NS | NS | 0.20 | 0.20 | 0.1 | NS |
| **Ar** | 403.88 | 54.06 | 107.48 | 35.12 | 99.13 | 29.84 | 276.63 | 60.19 | 406.50 | 48.53 | <0.01 | <0.05 | <0.05 | NS | NS | NS | NS | <0.05 | NS | NS | NS |
| **Rarα** | 187.85 | 15.03 | 131.26 | 13.06 | 41.55 | 3.30 | 183.25 | 33.53 | 176.93 | 6.20 | <0.01 | NS | <0.01 | NS | NS | NS | NS | NS | 0.04 | <0.05 | NS |
| **Shp** | 6590.78 | 907.36 | 1383.62 | 294.80 | 3011.60 | 373.80 | 5439.80 | 1322.31 | 5229.30 | 452.83 | <0.01 | <0.01 | 0.20 | NS | NS | NS | <0.05 | <0.05 | NS | NS | NS |
| **Car** | 47795.90 | 5124.59 | 51846.60 | 6414.65 | 40458.08 | 6738.60 | 14310.33 | 4243.04 | 21042.20 | 1911.85 | <0.01 | NS | NS | <0.05 | NS | NS | <0.05 | 0.09 | 0.20 | NS | NS |
| **Rarβ** | 70.83 | 15.22 | 37.34 | 4.98 | 17.55 | 4.38 | 101.30 | 29.58 | 152.83 | 54.66 | <0.01 | NS | 0.20 | NS | NS | NS | NS | 0.20 | 0.03 | <0.01 | NS |
| **Rxrβ** | 1729.28 | 125.73 | 1003.60 | 63.29 | 592.25 | 100.35 | 1326.60 | 277.45 | 1333.75 | 122.60 | <0.01 | NS | <0.01 | NS | NS | NS | NS | NS | 0.13 | 0.08 | NS |
| **Nur-77** | 885.05 | 275.56 | 204.08 | 61.63 | 209.80 | 89.61 | 222.13 | 37.59 | 55.90 | 10.52 | <0.01 | NS | 0.20 | NS | <0.01 | NS | NS | NS | NS | NS | NS |
| **Rorγ** | 2404.95 | 143.21 | 2357.74 | 302.34 | 913.58 | 82.01 | 1343.70 | 419.52 | 2917.13 | 188.87 | <0.05 | NS | 0.20 | NS | NS | 0.20 | NS | NS | NS | <0.01 | NS |
| **Couptf1** | 28.65 | 4.17 | 14.90 | 2.67 | 24.85 | 3.34 | 39.30 | 4.95 | 31.83 | 3.32 | <0.05 | NS | NS | NS | NS | NS | <0.01 | <0.05 | NS | NS | NS |
| **Gr** | 2334.90 | 143.76 | 953.90 | 97.64 | 776.23 | 175.14 | 1516.38 | 495.45 | 1925.00 | 224.16 | <0.05 | <0.05 | 0.01 | NS | NS | NS | NS | NS | NS | 0.2 | NS |
| **Errγ** | 71.10 | 9.55 | 4.06 | 1.72 | 9.05 | 3.15 | 11.33 | 3.62 | 11.85 | 3.24 | <0.05 | <0.01 | 0.20 | NS | NS | NS | NS | NS | NS | NS | NS |
| **Tr4** | 1821.45 | 47.85 | 1344.52 | 186.87 | 718.18 | 154.31 | 1709.98 | 136.96 | 1944.33 | 243.66 | <0.05 | NS | <0.05 | NS | NS | NS | NS | NS | 0.20 | <0.05 | NS |
| **Rxrγ** | 61.63 | 5.51 | 20.02 | 2.64 | 20.10 | 8.06 | 17.35 | 8.17 | 30.95 | 2.67 | <0.05 | <0.05 | <0.05 | <0.05 | NS | NS | NS | NS | NS | NS | NS |
| **Hnf4γ** | 55954.25 | 2715.89 | 55941.12 | 604.07 | 38719.55 | 8761.20 | 72964.58 | 4096.17 | 57423.75 | 4058.95 | <0.05 | NS | NS | NS | NS | NS | NS | NS | <0.01 | NS | NS |
| **Nor-1** | 0.73 | 0.27 | 3.22 | 0.59 | 3.48 | 1.15 | 5.55 | 0.95 | 3.35 | 0.89 | <0.05 | NS | 0.20 | <0.01 | NS | NS | NS | NS | NS | NS | NS |
| **Fxrα** | 2252.60 | 240.36 | 1400.06 | 548.35 | 1236.45 | 95.84 | 1093.13 | 299.51 | 2862.28 | 304.67 | <0.05 | NS | NS | NS | NS | NS | NS | NS | NS | 0.08 | 0.07 |
| **Couptf2** | 6213.93 | 342.47 | 6673.00 | 236.13 | 3734.00 | 305.00 | 5781.70 | 306.65 | 5940.98 | 395.75 | <0.05 | NS | <0. 05 | NS | NS | NS | NS | NS | NS | NS | NS |
| **Lxrα** | 41344.00 | 2326.21 | 33961.18 | 1399.16 | 27118.48 | 2344.31 | 36561.23 | 2033.54 | 34543.45 | 2518.48 | <0.05 | NS | <0.01 | NS | NS | NS | NS | NS | NS | NS | NS |
| **Pparγ** | 379.40 | 66.90 | 205.46 | 30.57 | 140.93 | 35.73 | 163.18 | 36.82 | 181.08 | 22.07 | <0.05 | NS | <0.01 | 0.08 | NS | NS | NS | NS | NS | NS | NS |
| **Erα** | 142.95 | 15.34 | 132.74 | 33.17 | 97.75 | 21.63 | 215.95 | 62.81 | 288.60 | 33.72 | <0.05 | NS | NS | NS | NS | NS | NS | 0.20 | NS | <0.05 | NS |
| **Tr2** | 403.25 | 29.90 | 379.12 | 35.13 | 182.05 | 44.39 | 357.70 | 48.23 | 316.88 | 26.50 | <0.05 | NS | <0.05 | NS | NS | 0.10 | NS | NS | 0.20 | NS | NS |
| **Lrh1** | 23067.18 | 2606.45 | 13685.42 | 1526.73 | 16770.45 | 4424.85 | 25049.68 | 4070.25 | 21408.15 | 2395.49 | 0.07 | NS | NS | NS | NS | NS | NS | NS | NS | NS | NS |
| **Rxrα** | 49861.68 | 1394.63 | 40872.52 | 7169.07 | 28702.63 | 3379.38 | 35094.00 | 2596.27 | 37449.88 | 2552.84 | 0.09 | NS | <0.05 | NS | NS | NS | NS | NS | NS | NS | NS |
| **Hnf4α** | 778282.65 | 51354.68 | 700290.52 | 121050.91 | 431466.55 | 96515.30 | 624941.63 | 88458.67 | 615679.10 | 50542.47 | 0.20 | NS | 0.07 | NS | NS | NS | NS | NS | NS | NS | NS |
| **Pxr** | 19516.88 | 2374.67 | 13888.90 | 1098.41 | 15032.28 | 1959.49 | 23305.85 | 4783.60 | 17340.70 | 2994.81 | 0.30 | NS | NS | NS | NS | NS | NS | NS | NS | NS | NS |

**Table S2. Genes modulated by PPARδ activation in proliferating Hepa 1-6 cells in vitro**

| **SYMBOL** | **DMSO-Mean** | **DMSO - SD** | **GW-Mean** | **GW-SD** | **Ratio** | **P** | **CHRM** | **DEFINITION** | **SYNONYMS** |
| --- | --- | --- | --- | --- | --- | --- | --- | --- | --- |
| Pdk4 | 254.7 | 25.0 | 579.4 | 52.2 | 2.3 | 3.68E-38 | 6 | Mus musculus pyruvate dehydrogenase kinase, isoenzyme 4 (Pdk4) | AV005916 |
| Acot1 | 407.7 | 57.0 | 724.3 | 122.8 | 1.8 | 1.26E-06 | 12 | Mus musculus acyl-CoA thioesterase 1 (Acot1) | CTE-I; ACH2; Cte1; D12Ucla1; CTE-1 |
| Prickle1 | 497.0 | 94.4 | 811.9 | 113.2 | 1.6 | 2.12E-05 |  | Mus musculus prickle like 1 (Drosophila) (Prickle1) | Prickle; 1110058P22Rik; AW215793 |
| Angptl4 | 1734.4 | 224.4 | 2713.4 | 312.4 | 1.6 | 4.90E-06 | 17 | Mus musculus angiopoietin-like 4 (Angptl4) | HFARP; Bk89; PGARG; PGAR; pp1158; ARP4; Ng27; FIAF |
| Acaa2 | 416.6 | 48.0 | 631.3 | 98.5 | 1.5 | 0.000231727 | 18 | Mus musculus acetyl-Coenzyme A acyltransferase 2 (mitochondrial 3-oxoacyl-Coenzyme A thiolase) (Acaa2) | AI255831; D18Ertd240e; AI265397; 0610011L04Rik |
| Etfdh | 461.6 | 54.7 | 668.7 | 114.6 | 1.4 | 0.01662967 | 3 | Mus musculus electron transferring flavoprotein, dehydrogenase (Etfdh) | AV001013; 0610010I20Rik |
| Tsg101 | 582.4 | 56.9 | 835.9 | 198.8 | 1.4 | 0.01537338 | 7 | Mus musculus tumor susceptibility gene 101 (Tsg101) | CC2; AI255943 |
| Cyp51 | 830.3 | 130.0 | 1186.0 | 304.6 | 1.4 | 0.03187813 | 5 | Mus musculus cytochrome P450, family 51 (Cyp51) | AI426508 |
| Cct5 | 729.8 | 62.3 | 1041.9 | 292.1 | 1.4 | 0.03992423 | 15 | Mus musculus chaperonin containing Tcp1, subunit 5 (epsilon) (Cct5) | mKIAA0098; Ccte |
| Dgat2 | 226.7 | 24.8 | 321.3 | 36.2 | 1.4 | 4.27E-05 | 7 | Mus musculus diacylglycerol O-acyltransferase 2 (Dgat2) | DGAT-2; 0610010B06Rik |
| Hsd17b12 | 1678.3 | 120.6 | 2295.1 | 423.6 | 1.4 | 0.008576292 | 2 | Mus musculus hydroxysteroid (17-beta) dehydrogenase 12 (Hsd17b12) | KIK-I; Kik1; 2610510O05Rik; AI172963 |
| Thumpd3 | 761.6 | 79.5 | 1041.0 | 177.1 | 1.4 | 0.004645689 | 6 | Mus musculus THUMP domain containing 3 (Thumpd3) | AW556087; Gt(ROSA)26asSor; Gtrosa26as |
| Tmem59 | 1282.4 | 126.6 | 1731.7 | 180.4 | 1.4 | 0.001078933 | 4 | Mus musculus transmembrane protein 59 (Tmem59) | AI256529; 3110046P06Rik; 1110001M20Rik; MTDCF1; D4Ertd20e; ORF18 |
| Cd82 | 904.7 | 41.3 | 1220.9 | 243.7 | 1.3 | 0.01719099 |  | Mus musculus CD82 antigen (Cd82) | C33; Kai1; AL023070; Tspan27; AA682076 |
| Acadl | 388.6 | 14.3 | 523.7 | 26.9 | 1.3 | 0.004021676 | 1 | Mus musculus acyl-Coenzyme A dehydrogenase, long-chain (Acadl) | AA960361; LCAD; C79855; AU018452 |
| Metap2 | 417.1 | 27.9 | 558.5 | 92.9 | 1.3 | 0.02956687 | 10 | Mus musculus methionine aminopeptidase 2 (Metap2) | AU014659; Amp2; AI047573; Mnpep; A930035J23Rik; p67eIF2; MGC102452; AL024412; p67 |
| Ifrd1 | 325.2 | 25.6 | 426.9 | 69.0 | 1.3 | 0.008283781 | 12 | Mus musculus interferon-related developmental regulator 1 (Ifrd1) | Ifnl; PC4; Tis7 |
| Pdss1 | 315.2 | 30.4 | 413.0 | 42.3 | 1.3 | 0.000601764 | 2 | Mus musculus prenyl (solanesyl) diphosphate synthase, subunit 1 (Pdss1) | Tprt; TPT; mDLP1; mSPS1; 2610203G20Rik; 2700031G06Rik |
| Lpcat3 | 1321.0 | 149.2 | 1729.2 | 214.4 | 1.3 | 0.00423273 | 6 | Mus musculus lysophosphatidylcholine acyltransferase 3 (Lpcat3) | Oact5; C3f; Moact5; PTG; Grcc3f |
| Smox | 760.9 | 38.1 | 994.6 | 118.5 | 1.3 | 0.004960764 | 2 | Mus musculus spermine oxidase (Smox) | PAO; PAOh1; B130066H01Rik; SMO |
| Memo1 | 361.7 | 25.2 | 472.2 | 67.6 | 1.3 | 0.004517887 | 17 | Mus musculus mediator of cell motility 1 (Memo1) | 0610016J10Rik; D930048L02Rik |
| Prune | 551.5 | 64.4 | 719.4 | 109.9 | 1.3 | 0.03569194 | 3 | Mus musculus prune homolog (Drosophila) (Prune) | Prune-M1; DRES-17; HTCD37; 9230112O05Rik; C130058A12; PRUNEM1 |
| Chmp5 | 487.6 | 52.8 | 634.8 | 72.6 | 1.3 | 0.00178862 | 4 | Mus musculus chromatin modifying protein 5 (Chmp5) | RP23-28I8.4; AW545668; 2210412K09Rik |
| Lpl | 926.7 | 176.5 | 1201.0 | 119.3 | 1.3 | 0.01983367 | 8 | Mus musculus lipoprotein lipase (Lpl) |  |
| Pgrmc1 | 849.5 | 113.2 | 1099.3 | 132.5 | 1.3 | 0.005915439 | X | Mus musculus progesterone receptor membrane component 1 (Pgrmc1) | HPR6.6; PPMR; AA415812; Vema |
| Vbp1 | 611.0 | 39.0 | 790.3 | 161.4 | 1.3 | 0.04091804 | X | Mus musculus von Hippel-Lindau binding protein 1 (Vbp1) |  |
| Ecm1 | 3286.4 | 353.5 | 4231.8 | 1242.8 | 1.3 | 0.04631307 | 3 | Mus musculus extracellular matrix protein 1 (Ecm1) | p85; AI663821 |
| Nudcd2 | 666.9 | 91.0 | 854.6 | 8.4 | 1.3 | 0.007927175 | 11 | Mus musculus NudC domain containing 2 (Nudcd2) | D11Ertd603e; RP23-382C18.4; 2700047N05Rik |
| Elovl6 | 521.1 | 76.5 | 667.4 | 121.9 | 1.3 | 0.04320862 | 3 | Mus musculus ELOVL family member 6, elongation of long chain fatty acids (yeast) (Elovl6) | MGC107467; FAE; C77826; LCE |
| Ankrd46 | 771.9 | 93.8 | 983.0 | 177.2 | 1.3 | 0.03568605 | 15 | Mus musculus ankyrin repeat domain 46 (Ankrd46) | 1110054N06Rik; AI987733; AI314978 |
| Casp8 | 1061.1 | 149.0 | 1346.5 | 229.5 | 1.3 | 0.03590499 | 1 | Mus musculus caspase 8 (Casp8), transcript variant 2 | MACH; FLICE; Caspase-8; Mch5 |
| Fcgrt | 893.8 | 105.4 | 1132.6 | 149.0 | 1.3 | 0.009105871 | 7 | Mus musculus Fc receptor, IgG, alpha chain transporter (Fcgrt) | FcRn |
| Tfam | 785.1 | 51.9 | 991.7 | 70.0 | 1.3 | 0.009913767 |  | Mus musculus transcription factor A, mitochondrial (Tfam), nuclear gene encoding mitochondrial protein | Hmgts; mtTFA; tsHMG; AI661103 |
| Idi1 | 325.3 | 35.6 | 407.7 | 36.5 | 1.3 | 0.005533073 | 13 | Mus musculus isopentenyl-diphosphate delta isomerase (Idi1), transcript variant 2 | MGC118013; 4832416K17Rik; MGC8139 |
| Dazap2 | 3615.1 | 345.8 | 4529.4 | 552.0 | 1.3 | 0.01622341 | 15 | Mus musculus DAZ associated protein 2 (Dazap2) | Brbp; AI314727; mKIAA0058; MGC25222; Prtb; gt6-12 |
| Arid5b | 541.5 | 75.4 | 405.6 | 59.8 | 0.7 | 0.00523621 | 10 | Mus musculus AT rich interactive domain 5B (MRF1-like) (Arid5b) | Mrf2; Mrf2alpha; AI467247; 4930580B11; Mrf2beta; Desrt; 5430435G07Rik |
| Dusp7 | 1824.1 | 318.0 | 1363.7 | 94.8 | 0.7 | 0.01153452 | 9 | Mus musculus dual specificity phosphatase 7 (Dusp7) | MKPX; MKP-X; AU015694; PYST2 |
| BC017647 | 1481.8 | 227.9 | 1104.1 | 111.3 | 0.7 | 0.004955167 | 11 | Mus musculus cDNA sequence BC017647 (BC017647) | MGC28990 |
| Zc3h18 | 1631.1 | 288.4 | 1207.7 | 103.5 | 0.7 | 0.009891912 | 8 | Mus musculus zinc finger CCCH-type containing 18 (Zc3h18), transcript variant 1 | 1190001B23Rik; Nhn1 |
| Nfatc3 | 434.1 | 66.1 | 320.3 | 37.1 | 0.7 | 0.003190464 | 8 | Mus musculus nuclear factor of activated T-cells, cytoplasmic, calcineurin-dependent 3 (Nfatc3) | NFAT4; D8Ertd281e; NFATx; C80703 |
| Tcof1 | 2241.0 | 457.3 | 1640.7 | 272.1 | 0.7 | 0.0241333 | 18 | Mus musculus Treacher Collins Franceschetti syndrome 1, homolog (Tcof1) | AW209012; treacle; AA408847 |
| Mical2 | 733.0 | 106.9 | 533.2 | 95.7 | 0.7 | 0.005586787 | 7 | Mus musculus microtubule associated monoxygenase, calponin and LIM domain containing 2 (Mical2) | KIAA0750; mKIAA0750; 9530064J02; 5330438E18Rik |
| Pold1 | 2504.5 | 613.3 | 1819.1 | 332.4 | 0.7 | 0.04947108 | 7 | Mus musculus polymerase (DNA directed), delta 1, catalytic subunit (Pold1) | 125kDa |
| Cacna2d1 | 365.8 | 15.9 | 265.2 | 35.7 | 0.7 | 0.000142427 | 5 | Mus musculus calcium channel, voltage-dependent, alpha2/delta subunit 1 (Cacna2d1) | MGC141430; Cchl2a; Cacna2 |
| Pcyt1a | 597.0 | 60.8 | 426.0 | 54.9 | 0.7 | 7.73E-05 | 16 | Mus musculus phosphate cytidylyltransferase 1, choline, alpha isoform (Pcyt1a) | CTalpha; Cttalpha; Ctpct |
| Zbtb38 | 400.5 | 39.7 | 268.5 | 42.3 | 0.7 | 1.22E-05 | 9 | Mus musculus zinc finger and BTB domain containing 38 (Zbtb38) | A930014K01Rik; CIBZ |
| Zbtb7a | 647.7 | 12.3 | 415.7 | 57.8 | 0.6 | 6.54E-07 | 10 | Mus musculus zinc finger and BTB domain containing 7a (Zbtb7a) | AI452336; Lrf; Pokemon; Zbtb7; 9130006G12Rik; FBI-1; 9030619K07Rik |
| Bach1 | 475.1 | 45.4 | 278.9 | 50.4 | 0.6 | 6.16E-08 | 16 | Mus musculus BTB and CNC homology 1 (Bach1) | 6230421P05Rik; AI323795 |
